# A method for calculating left ventricular end-diastolic volume as an index of left ventricular preload from the pre-ejection period, ejection time, blood pressure, and stroke volume: a prospective, observational study

**DOI:** 10.1186/s12871-023-02103-2

**Published:** 2023-04-28

**Authors:** Mitsuyo Hayabuchi, Yuka Matsuki, Shuhei Kidoguchi, Kenji Shigemi

**Affiliations:** 1 Department of Anesthesiology and Reanimatology, University of Fukui Hospital, Fukui, Japan; 2 Faculty of Medicine Sciences, Department of Anesthesiology & Reanimatology, University of Fukui, 23-3 Eiheijicho, Yoshidagun, Fukui, 910-1193 Japan; 3 Department of Clinical Laboratory, University of Fukui Hospital, Fukui, Japan

**Keywords:** Left ventricular end diastolic volume, Ees/Ea, Stroke volume

## Abstract

**Background:**

Left ventricular end-diastolic volume (EDV) is a major determinant of cardiac preload. However, its use in fluid management is limited by the lack of a simple means to measure it noninvasively. This study presents a new noninvasive method that was validated against simultaneously measured EDV by transthoracic echocardiography (TTE). The goal of this study was to develop and validate a method to estimate EDV in humans non-invasively from left ventricular arterial coupling (Ees/Ea) and stroke volume (SV).

**Methods:**

Ees/Ea can be calculated non-invasively from the four parameters of end-systolic arterial pressure (Pes), diastolic arterial pressure (DBP), pre-ejection period (PEP), and ejection time (ET), using the approximation formula. In addition, if SV can be assessed, EDV can be calculated. Therefore, using a vascular screening system (VaSera 1000/1500, Fukuda Denshi Co., Ltd., Tokyo, Japan), blood pressure, PEP, and ET were measured noninvasively, the SV value was obtained using an ultrasound diagnostic device, EDV was calculated (EDV calc), and it was compared with EDV obtained using the ultrasound diagnostic device (EDV echo). The results are shown as mean ± standard deviation values.

**Results:**

There were 48 healthy subjects (40 men, 8 women), with a mean age of 24 ± 4 years, mean height of 169 ± 7 cm, and mean weight of 65 ± 12 kg. EDV echo was 91 ± 16 ml, and EDV calc was 102 ± 21 ml. There was a significant correlation between EDV echo and EDV calc (*R*^2^ = 0.81, *p* < 0.01). A Bland–Altman plot between EDV echo and EDV calc showed that the bias and limits of agreement were –11.2 ml (-36.6, + 14.2 ml).

**Conclusions:**

The results suggest that EDV can be measured non-invasively from Ees/Ea and SV. This suggests that continuous measurements may potentially work, using equipment available in the intraoperative setting.

## Background

The goal of fluid management is to maintain adequate end organ perfusion by optimizing stroke volume (SV) and cardiac output. SV depends on cardiac preload, afterload, and myocardial contractility. Therefore, preload assessment is important to determine the adequate fluid amount. Left ventricular end-diastolic volume (EDV) is a surrogate measure for cardiac preload. It has been reported that left ventricular end-diastolic area measured using transesophageal echocardiography is a reliable predictor of cardiac preload in patients with early septic shock [1]. However, the limitation of an ultrasound-based method is that it cannot perform continuous monitoring, and it relies on the proper acquisition and interpretation of the results. Currently, there is no clinical monitoring method to assess EDV noninvasively and continuously.

Left ventricular pressure–volume relationships have been used to understand cardiac mechanics. End-systolic and end-diastolic pressure–volume relationships (ESPVR and EDPVR) describe left ventricular systolic and diastolic properties, respectively. Ventricular-arterial coupling (V-A coupling), which is estimated from pressure volume loops, is expressed as the ratio of the end-systolic elastance (Ees) and the effective arterial elastance (Ea). Ees is the slope of the line connecting the end-systolic pressures (Pes) and volumes (Ves), such that Ees = Pes / (Ves—V0), where V0 is the volume at zero intraventricular pressure. Ea is defined as the slope of the line connecting EDV on the volume axis to the end-systolic pressure volume point. Ea is the ratio between Pes and stroke volume. According to the definition of V-A coupling, EDV can be calculated from Ees/Ea and stroke volume.

A previous study introduced a noninvasive estimation of Ees/Ea using four parameters, namely pre-ejection period (PEP), ejection time (ET), end-systolic pressure (Pes), and diastolic pressure (Pd) [2]. This method enabled us to estimate Ees/Ea beat to beat, because these four parameters can be obtained continuously using an electrocardiogram, pulse waveform, and phonocardiogram. In this study, these four parameters (PEP, ET, Pad, and Pes) were measured continuously and noninvasively. PEP and ET were obtained from the patient’s electrocardiogram, pulse waveform, and phonocardiogram. Pad and Pes were obtained from a pulse wave-measuring device (VaSera 1000/1500, Fukuda Denshi Co., Ltd., Tokyo, Japan). In the future, this method, in combination with SV using a pulse wave cardiac output monitor, might be useful for continuous, minimally invasive monitoring of EDV in real time during general anesthesia. The aim of the present study was to estimate EDV using V-A coupling (Ees/Ea) in healthy subjects.

## Methods

### Study population

This was a prospective, observational study. The study protocol was approved by the Research Ethics Committee of the University of Fukui Hospital (No. 20140125) and was conducted in accordance with the Declaration of Helsinki. Fifty-eight healthy adult subjects were studied at the University of Fukui Hospital between 2018 and 2020. Subjects with a history of cardiovascular diseases were excluded. Informed consent was obtained from all participants and/or their legal guardian(s).

### Measurements

The age, sex, height, and weight of the subjects were recorded. Each subject was placed in the left lateral position, and transthoracic echocardiography was performed using the Vivid E9 (GE Healthcare Japan, Tokyo, Japan). All acquisitions were performed by a single examiner. The examiner was certified as a sonographer by the Japanese Society of Ultrasound Medicine and had at least three years of work experience.

Left ventricular end-systolic volume and EDV were obtained by the modified Simpson method using the apical 4-lm and 2-lm images. SV was estimated by subtracting the left ventricular end-systolic volume from the left ventricular EDV.

**Fig. 1:**
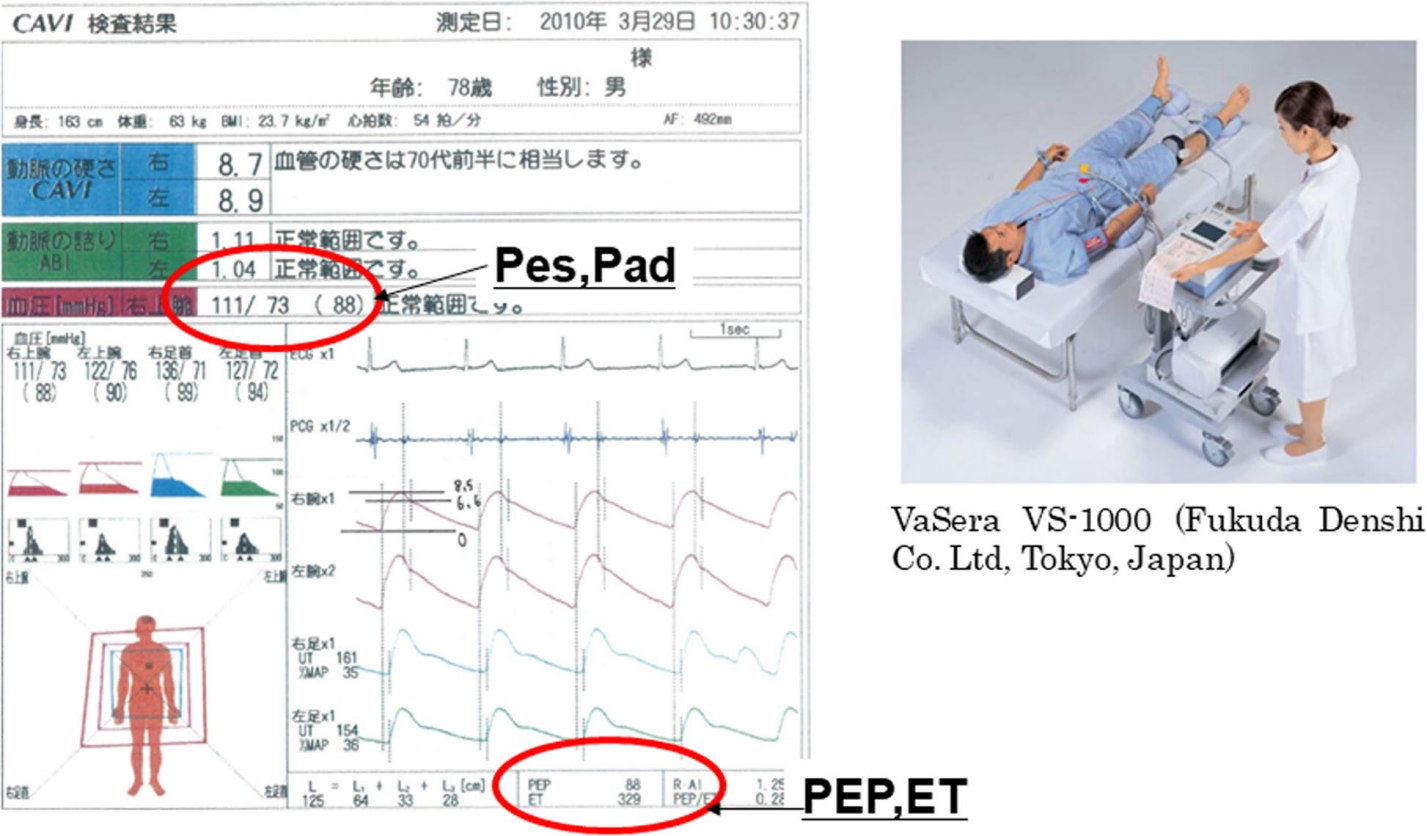
Cardio-ankle vascular index. Four parameters (PEP, ET, Pad, and Pes) are obtained from a pulse wave measuring device (VaSera 1000/1500) continuously. PEP and ET are obtained from the patient’s electrocardiogram, pulse waveform, and phonocardiogram. Pad and Pes are obtained from a pulse wave-measuring device (VaSera)

Each subject was then placed in the supine position. A standard four-lead ECG was attached, and a stethoscope was attached and connected to the main unit, which was placed on the sternal border at the second intercostal space. Four blood pressure cuffs were placed around the arms and ankles. The subjects’ ECG, phonocardiogram, brachial arterial pulse waves, and tibial arterial pulse waves were simultaneously recorded using the vascular screening system, VaSera 1000/1500 (Fig. 1). The pre-ejection period (PEP), ejection time (ET), systolic blood pressure (SBP), and diastolic blood pressure (DBP) were automatically recorded by the device. ET was defined as the time difference between the upstroke and the dicrotic notch of the right brachial pulse wave. PEP was obtained by subtracting ET from the time between the Q wave and the second heart sound. SBP and DBP were determined by cuff sphygmomanometry. End-systolic pressure (Pes) was calculated from SBP and DBP using the formula published by Kappus et al [3]:

1 $$\mathrm{Pes }= (0.205\times \mathrm{SBP}) + (0.898\times \mathrm{DBP}) + 0.4214.$$

Then, ventricular-arterial coupling (the ratio of end-systolic elastance to arterial elastance) was calculated using the values of PEP, ET, Pes, and DBP, as described previously [2]. When left ventricular elastance E(t) is approximated bilinearly with the isovolumic contraction and ejection periods, the resultant slope ratio (velocity ratio of the decrease in left ventricular elastance) is defined as k. For bilinear elastance approximation, isovolumetric systole and ejection period are approximated with their respective lines. Diastole is unrelated, and the overall heart rate is not involved.

The hypothetical left ventricular end-systolic pressure when the aorta is clamped, such that the ventricle does not eject into the arteries (peak isovolumic pressure; Pmax), is expressed as below using PEP, ET, and Pad.

2 $$\mathrm{Pmax }=\mathrm{ Pad }+\mathrm{ Pad }(\mathrm{ET }/\mathrm{ PEP})\mathrm{ k }=\mathrm{ Pad }[1 + (\mathrm{ ET }/\mathrm{ PEP})\mathrm{ k}]$$

Meanwhile, since the increase in left ventricular pressure from Pes to Pmax, and the increase in aortic pressure to Pes are attributable to the same ventricular ejection volume, the ratio of Ees to Es can be expressed as

3 $$\mathrm{Ees }/\mathrm{ Ea }= (\mathrm{ Pmax}-\mathrm{Pes}) /\mathrm{ Pes}$$

**Fig. 2:**
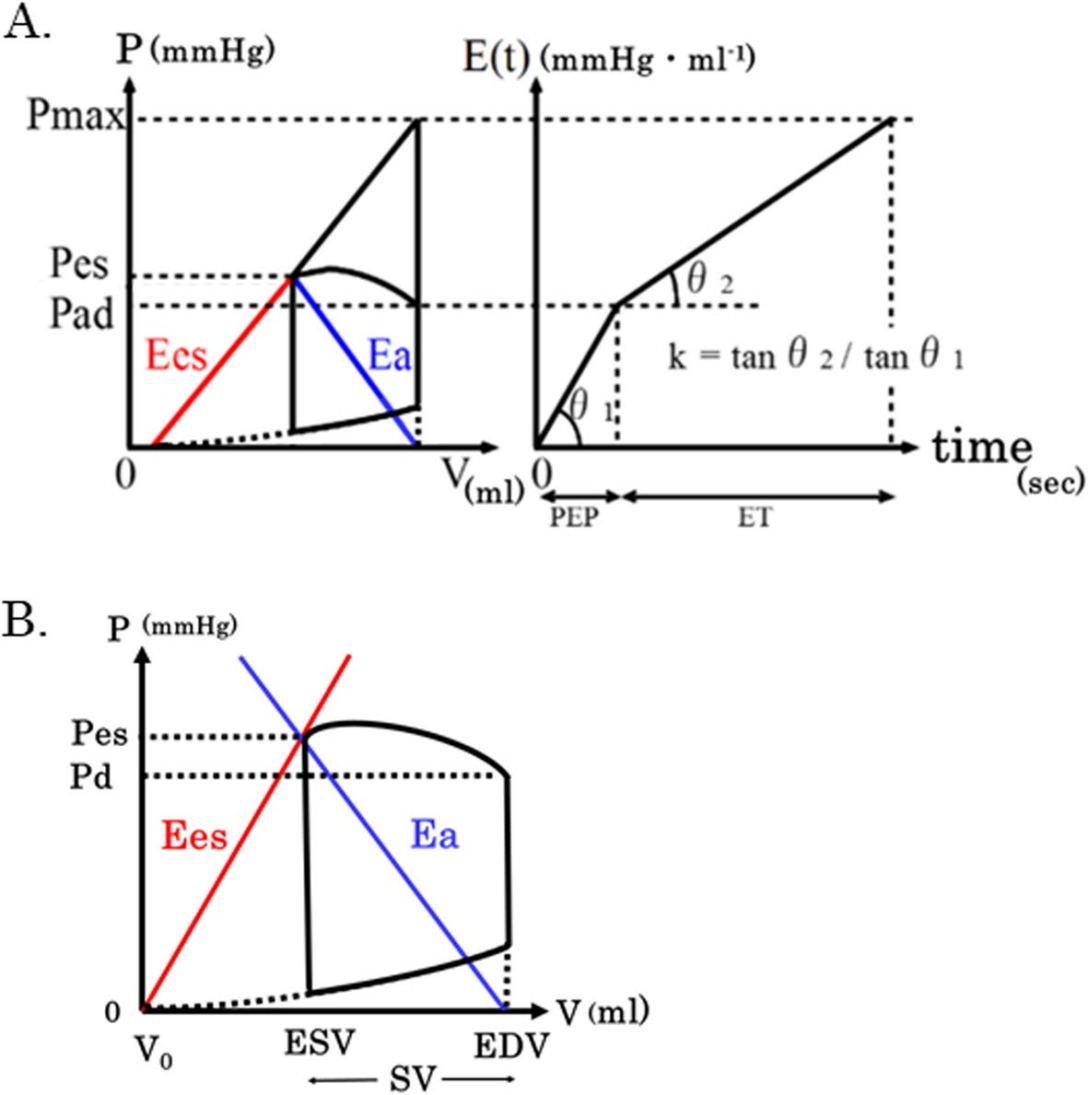
Equation for estimating EDV. **A** Schematic diagram of a pressure–volume loop, end-systolic pressure–volume relationship (slope of end-systolic elastance [Ees]), arterial pressure–volume relationship (negative slope of effective arterial elastance [Ea]), end-systolic pressure (Pes), ventricular pressure at which the ventricle begins to eject (Pad), and putative isovolumic pressure (Pmax) (left). The figure on the right shows the left ventricular elastance (E(t)) of systole only, approximated with lines for the pre-injection period (PEP) and ejection time (ET), respectively. The ratio of the slopes of those two lines (tanθ1/tanθ2) is expressed as a constant (k) (right). See reference [1] for details. **B** Trace of the left ventricular volume of one heartbeat. The y-axis shows pressure, and the x-axis shows volume. In the figure, this is expressed as Ea = Pes/SV, Ees = Pes/(ESV-V_0_) = Pes/(EDV-SV-V_0_). From Ees/Ea = SV/(EDV-SV-V_0_), this becomes EDV = SV (1 + Ea/Ees) + V_0_. It was assumed that V_0_ is equal to zero. Therefore, EDV = SV (1 + Ea/Ees)

From Eqs. (2) and (3), the theoretical equation

4 $$\mathrm{Ees }/\mathrm{ Ea }=\mathrm{ Pad }/\mathrm{ Pes}\bullet (1 +\mathrm{ k}\bullet \mathrm{ET }/\mathrm{ PEP})-1$$

is derived. Thus, if the value of k is known, the coupling value (Ees/Ea) can be calculated as shown in Eq. (4), using arterial pressure and systolic time measurements, without measuring left ventricular pressure–volume. If this theoretical equation and the experimentally obtained

5 $$\mathrm{k }= 0.53 {(\mathrm{Ees}/\mathrm{Ea})}^{0.51}$$

are simultaneously set up and solved by Newton’s method, Ees/Ea is obtained (Fig. 2A).

Left ventricular end-systolic elastance (Ees) is defined by the following equation,

6 $$\mathrm{Ees }=\mathrm{ Pes }/ (\mathrm{EDV }-\mathrm{ SV }-\mathrm{ Vo})$$

where Vo is the volume at a Pes of 0 mmHg. It was assumed that Vo is equal to 0 ml in this study. Arterial elastance is defined using the formula published by Sunagawa et al., [4] as follows,

7 $$\mathrm{Ea }=\mathrm{ Pes }/\mathrm{ SV}$$

Solving Eqs. (4) and (5) enables EDV (EDV calc) to be estimated from SV and Ea/Ees (Fig. 2B).

8 $$\mathrm{EDV calc }=\mathrm{ SV }(1 +\mathrm{ Ea}/\mathrm{Ees})$$

SV in EDV calc and EDV echo were measured using the modified Simpson method.

### Statistical analysis

JMP13 was used for the analysis. Continuous variables are reported as average and standard deviation (SD) values, and categorical variables are reported as proportions. Simple linear regression was used to compare EDV echo and EDV calc. Then, Bland–Altman analysis was used to assess agreement between the two. The bias (the mean difference between EDV echo and EDV calc), limits of agreement, and percentage error (PE) were calculated. The percentage error was calculated using the formula, PE = 2 SD of bias/mean value of the reference method. The agreement between EDV calc and EDV echo was interpreted as clinically acceptable when the PE was 30% or less [5].　A *p*-value less than 0.05 was considered significant.

## Results

**Fig. 3:**
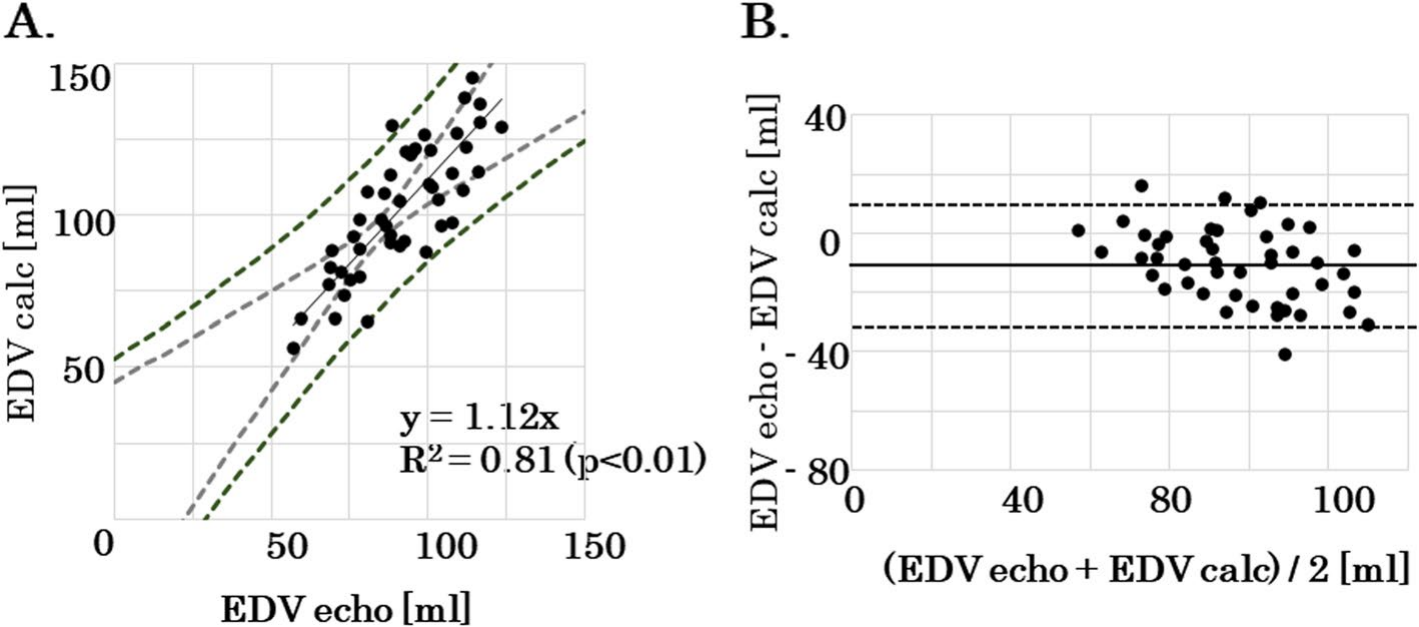
**A** Correlation between EDV echo and EDV calc. The correlation between EDV echo and EDV calc is significant (*R*^2^ = 0.81, *p* < 0.01). **B** Bland–Altman plot between EDV echo and EDV calc. The bias ± standard deviation is –11.2 ± 12.7 ml

The study included 58 healthy adults. Of them, adults with missing data or SV ≥ 140 ml were excluded, and the analysis was done with the remaining 48 healthy subjects. The subjects’ background characteristics are shown in Table 1. The subjects were 40 men and 8 women, with a mean age of 24 ± 4 years, mean height of 169 ± 7 cm, and mean weight of 65 ± 12 kg. EDV echo was 91 ± 16 ml, and EDV calc was 102 ± 21 ml. There was a significant correlation between EDV echo and EDV calc (*R*^2^ = 0.81, *p* < 0.01) (Fig. 3A). The bias ± standard deviation was –11.2 ± 12.7 ml. The limits of agreement were –36.6 to 14.2 ml (Fig. 3B). The percentage error for EDV calc was 27.7%.

**Table 1:** Main characteristics of the patients (*n* = 48)

Characteristic	Value
Age (range), y	24 ± 4
Sex, male/ female, %	83% / 17%
Height, cm	169 ± 7
Weight, kg	65 ± 12
BMI, kg/m^2^	23 ± 3
SBP, mmHg	117 ± 13
DBP, mmHg	73 ± 9
PEP, second	96 ± 13
ET, second	286 ± 77
EDV echo, ml	91 ± 16
EDV calc, ml	102 ± 21
SV, ml	61 ± 13

Data are presented as mean ± standard deviation values, except where otherwise indicated

*BMI* Body mass index, *SBP* Systolic blood pressure, *DBP* Diastolic blood pressure, *PEP* Pre-ejection period, *ET* Ejection time, *EDV* Left ventricular end-diastolic volume, *SV* Stroke volume

## Discussion

In this study, EDV was determined from V-A coupling (Ees/Ea) measured non-invasively, and whether it could be used as a substitute for EDV measured by echocardiography was investigated. The results showed a significant correlation and compatibility between the two, and that EDV from Ees/Ea can be used as an alternative to EDV measured by echocardiography. The PE value supports the conclusion that EDV calc and EDV echo are almost in agreement.

A reliable estimation of cardiac preload is helpful in the management of severe circulatory dysfunction. The assessment of central venous pressure (CVP), pulmonary artery occlusion pressure (PAOP), pulmonary capillary wedge pressure (PCWP), and EDV indices as preload markers has been the mainstream of advanced hemodynamic monitoring for years [6]. EDV is a surrogate measure for cardiac preload. EDV can be directly measured with techniques such as cardiac computed tomography, real-time three-dimensional transesophageal echocardiography, or cardiac magnetic resonance imaging [7], but these techniques cannot be used intraoperatively. Today, stroke volume variation (SVV) and global end-diastolic volume have been commercialized as techniques to monitor cardiac preload [8, 9]. SVV is a value expressed as a rate of change of respiratory variability of stroke volume from arterial pressure waveform information of FloTrac™ (Edwards Lifesciences, Irvine, CA, USA).

However, though these measurements are non-invasive, they are not universally available and require the insertion of an invasive arterial pressure line. Therefore, since clinical monitoring for a more direct, non-invasive, and continuous assessment of EDV does not currently exist, SVV can be used as a surrogate measure for preload. This indicates that continuous measurements may potentially work, using equipment available in the intraoperative setting.

A clinically acceptable bias for ventricular volumes is often considered to be within 10 ml, and the bias found with this method was slightly greater than that, at 11.2 ml. However, we think this method is still clinically useful as long as there is correction for this bias, since it is constant [10]. The PEP and ET used in the present study could be noninvasively measured using a vascular screening system (VaSera VS-1000/1500). They can be measured using an oscillometric method with cuffs around both upper arms and both legs. Systolic blood pressure (Ps) and diastolic blood pressure (Pd) are also displayed. In addition, according to the study of Kappus et al., a substitution equation that uses Ps and Pd can be used for Ped [3]. The values of EDV (EDV calc) using a vascular screening system (VaSera VS-1000/1500) in the present study were compared with those measured (EDV echo) using the ultrasound device. The basic principle of this study was to calculate EDV by calculating Ees/Ea from PEP, ET, Pad, and Pes and adding SV.

PEP, ET, SBP, and DBP were measured in the supine position, whereas echocardiography was performed in the left lateral position. This is because it is easier to delineate the apex of the heart and obtain more accurate information on the apex in this position, which is necessary for the Simpson method. However, there is no difference in EDV between the supine and left lateral positions because there is no postural difference in venous return in healthy subjects [11].

Further study is needed to compare the utility of the EDV from the present method and SVV based on invasive arterial pressure waveform analysis. However, though with SVV the effects on SV of fluctuations in intrathoracic pressure from artificial respiration are quantified and monitored, the present method is an index that is unrelated to artificial respiration and so will have widespread clinical applications; for example, it can be used even in patients who are awake during intensive care.

SV measured with transthoracic ultrasonography was used in the present study. However, SV obtained from transthoracic echocardiography equipment can be replaced with SV obtained from arterial pressure-based cardiac output (APCO). In the future, it will be possible to measure EDV continuously by using a method that predicts SV noninvasively from estimated continuous cardiac output [12].

### Study limitations

The present study has several limitations. First, V0 was assumed to be 0 ml in the present method. Since this study included cases with normal cardiac function without heart failure, V0 was taken as zero [13, 14]. Second, the subjects in the present study were young, healthy people with normal cardiac function. In a past report [15], EDV in Japanese persons in their 20 s and 30 s was about 100 ml, and so in the present study, subjects with EDV ≥ 140 ml were excluded. The purpose of this study was not to accurately measure abnormal values, but to monitor whether EDV was maintained within the normal range. Therefore, subjects with a history of cardiovascular disease were excluded. In the future, it will be necessary to investigate whether there is a similar correlation and compatibility between EDV echo and EDV calc in patients with EDV ≥ 140 ml and patients with decreased cardiac function. The usefulness of this method for cardiovascular patients is under investigation. Third, both the SV used in the calculation and the EDV quoted as the measured value are data from the Simpson method. The same volumetric measurements are used for both measurements, which could lead to a significant bias in validation. Fully independent measurements of SV and EDV are needed to validate the methodology in the future. Therefore, we plan to use the SV obtained by other noninvasive continuous monitoring methods for the calculation instead of TTE in the future.

## Conclusions

The results suggest that continuous measurements may potentially work, using equipment available in the intraoperative setting.

## Data Availability

The datasets used and/or analyzed during the current study are available from the corresponding author on reasonable request.
